# Prescription Department

**Published:** 1892-12

**Authors:** 


IRRITABLE BLADDER WITH ACID URINE—

R. Spts. ammon. arom.,
Æther nit.,
Tr. hyoscyami, aa dr. ij.
Aq. camph., q. s., ad. oz. vi.

M. Sig. One oz. 3 times a day.

MAMMARY ABSCESS—TO PREVENT.—

R. Aquæ ferv., Oj.
Ammoniæ carb., oz. i.

M. S. Apply by means of cloths, as hot as can be borne, every 2 hours, or oftener.—*Dr. Miall.*

MUSCULAR RHEUMATISM.—

R. Ammon. chlorid., dr. i.
Ext. cimicifugæ Fl. oz. ij.
Syrupi,
Aquæ lauro-cerasi, aa oz. i.

M. Sig. Teaspoonful 3 or four times a day—*Prof. Bartholow.*

CHRONIC BRONCHITIS AND COUGH OF OLD AGE.—

R. Ammon. chlorid.,
Ext. glycyrrhiz, aa dr. i.
Spts. æther co., dr. ij.
Aquæ, ad. oz. vi.

Sig. Half oz. every 2 or 3 hours —*Dr. Waring.*

RHEUMATISM.—

R. Potassii iodidi, dr. iij.
Vini colchici seminis, oz. ij.
Tincturæ opii camphoratæ, oz. ij.
Tincturæ stramonii, dr. vi.
Tincturæ cemicifugæ, oz. iij.

M. S. Teaspoonful 3 or 4 times a day.

FOR EARACHE.—

R. Camphor chloral, dr. ij.
Glycerine, oz. iss.
Olei almond., oz. j.

M. Sig. A pledget of cotton is soaked in the drops aud introduced as far as possible into the ear.—*Prof. Pavesi.*

FOR OBSTINATE DIARRHŒA.—

R. Argenti nitratis, gr. ¼ ad ½.
Aqua destil., oz. ij.
Pulv. acaciæ, scruple ij.
Sacchari albi, dr. ij.

M. Sig. A teaspoonful or two every two hours.—*Dr. Constatt.*

Acute Bronchial Catarrh.—
R. Cinchoniæ sulph., dr. ss.
Ammon carb., gr. xv.
Camphoræ, gr. viij.
Morphiæ sulph., gr. ss.
M. Divide in capsule No. 4. Sig. One at bedtime.—*Daniel Lewis.*

Nervous or Sick Headache.—
R. Pot. bromid., gr. x.
Spts. ammon. arom., dr. ss.
Potass. cit. efferves., oz. i.
Sig. Take 3 times a day.—*Dr. Latham.*

Sciatica.—

R. Ol. Gaultheriæ,

Ol. terebinthinæ, aa dr. iv.

Syr. acaciæ, oz. ij.

Aq. Cassiæ, q. s ad. oz. iij.

M. S. Teaspoonful 3 or 4 times daily.—*Prof. Dana.*

Sprain Liniment.—
R. Olei terebinthinæ,
Acidi acetici, aa oz. ij.
Olei lavendulæ, dr. i.
Vitellum ovi.
Aquæ, ad oz. xvi.
M. S. Apply 2 or 3 times daily.

Acute Bronchitis.—
R. Potassi citratis, oz. j.
Syr. ipecacuanhæ, oz. j.
Succi limonis, oz. ij.
Aquæ, oz. iij.
M. Sig. Dr. ij every three hours. —By *Prof. H. C. Wood.*

Malignant Sore-throat.—
R. Ac. hydrochlor., dr. iss.
Decocti cinchon.
Inf. rosæ co , oz. iijss.
Mel. rosæ, oz. i.
M. Sig Gargle.—*Dr. Brande.*

For Impotency.—
R Ferri arseniatis, gr. v.
Extracta ergotæ aquosi, dr. ss.
Misce et fiant pil. No. xxx. Sig. Take one pill night and morning.—*Prof. Bartholow.*

Incontinence of Urine.—
R. Liq. strychniæ, B. P., mx.
Tr. belladonnæ, gtt. xx.
Inf. cascarillæ, ad. oz. ij.
M. Sig. Teaspoonful 3 times a day to a child 3 years old.—*Dr. Ellis.*

